# Electric and Magnetic Hotspots via Hollow InSb Microspheres for Enhanced Terahertz Spectroscopy

**DOI:** 10.1038/s41598-018-35833-2

**Published:** 2019-02-27

**Authors:** Mahdiyeh Sadrara, MirFaez Miri

**Affiliations:** Department of Physics, University of Tehran, P.O. Box 14395-547 Tehran, Iran

## Abstract

We study electric and magnetic hotspots in the gap between hollow InSb microspheres forming dimers and trimers. The outer radius, core volume fraction, distance, and temperature of the microspheres can be chosen to achieve field enhancement at a certain frequency corresponding to the transition between energy levels of a molecule placed in the gap. For example, utilizing 80 *μ*m radius spheres at a gap of 2 *μ*m held at a temperature of 295 K, allow electric field intensity enhancements of 10–2880 and magnetic field intensity enhancements of 3–61 in the frequency window 0.35–1.50 THz. The core volume fraction and the ambient temperature affect the enhancements, particularly in the frequency window 1.5–2 THz. Electric and magnetic hotspots are promising for THz absorption and circular dichroism spectroscopy.

## Introduction

In the past two decades, the generation and detection of coherent terahertz (THz) radiation have attracted much attention^1,2^. THz spectroscopy holds great promise for many applications in physics and chemistry^3,4^. The majority of studies exploit the Fourier transform spectroscopy and THz time-domain spectroscopy. In Fourier transform spectroscopy, the sample is illuminated with a broadband source. The sample is positioned in a Michelson interferometer and the path length of one of the arms of the interferometer is changed. The Fourier transform of the interference signal yields the absorption spectrum of the sample. In THz time-domain spectroscopy, these are the amplitude and phase of the electric field of THz pulses transmitted through the sample that determine its absorption and dispersion spectra.

THz spectroscopy has been used to study structure and dynamics of atoms and molecules. Many interesting results have been obtained, for instance: (i) Solute-induced changes in water’s hydrogen bonding network near lactose are investigated. It is found that the hydration layer around lactose extends to ≈5 Å from the surface corresponding to ≈123 water molecules beyond the first solvation shell^5^. (ii) Upon illumination by light, bacteriorhodopsin (BR) changes its conformation. It is observed that the photocycling time of the D96N mutant is about 1000 times that of the wild-type BR. The THz absorption of the mutant is smaller than that of the wild-type. This suggests that the mutant has lower conformational flexibility^6^. (iii) The low-frequency phonons of carbon nanotubes are directly observed^7^. (iv) The tunneling-inversion in methyl halides such as CH_3_Cl is demonstrated^8^.

Recently, the interest in THz circular dichroism spectroscopy is growing^9–11^. Unlike the absorption, the circular dichroism (the differential absorption of right- and left-circularly polarized light) is sensitive to the molecular chirality. Based on the abundance of biological chiral molecules, it is proposed that THz circular dichroism spectroscopy will be of use in searching for extraterrestrial life^9^. However, THz circular dichroism signals are extremely weak. This issue needs to be addressed.

In the visible and near-infrared regions of the spectrum, the spectroscopy techniques have been augmented with the use of *electric hotspots*^12–19^. Metallic nanoparticles supporting surface plasmons, concentrate light in small subwavelength volumes. Enhanced local electric fields significantly increase the interaction of light with atoms and molecules. In other words, hotspots may help to overcome the sensitivity limitations of conventional spectroscopy methods. Plasmonic enhanced fluorescence^15–17^, surface Raman optical activity of chiral molecules^18^, and surface Raman scattering^12^ are reported. Dielectric nanoparticles have recently gained attention due to the inherent ohmic losses of metallic nanoparticles which lead to heat perturbations on a nearby emitter^20,21^. Intense magnetic field in gaps separating dielectric nanoparticles are theoretically predicted^22^ and experimentally observed^23,24^. *Magnetic hotspots* also enhance the interaction of light with atoms and molecules: The basic Hamiltonian describing the interaction of an electromagnetic field and a quantum emitter is ${H}_{{\rm{int}}}=-\,{\bf{d}}\cdot {\bf{E}}-{\bf{m}}\cdot {\bf{B}}$ where **d** and **m** denote the electric and magnetic dipole moments of the emitter, respectively. Indeed ${\rm{Im}}({\bf{d}}\cdot {\bf{m}})\ne 0$ is a requisite for exhibiting circular dichroism^25^. Dielectric nanoparticles allow substantial increase of the circular dichroism signal^26,27^. Electric and magnetic hotspots provided by dimers and trimers of solid^28,29^ and hollow nanoparticles^30^ may find application in single molecule experiments. Hotspots provided by disordered and fractal aggregates of nanoparticles are also studied^31–35^. In particular, it is shown that fractal clusters of hollow silicon nanoparticles provide both electric and magnetic hotspots. In the wavelength window 400–750 nm, electric field intensity enhancements of 10–400 and magnetic field intensity enhancements of 10–3790 are reported^35^.

Taking advantage of the field enhancement for the spectroscopic studies, is beginning to be explored in the THz region of the spectrum^36–43^. For example, Park *et al*. utilized field enhancement within a nanoslit (a nanoaperture on a thin metallic film) to detect nanograms of molecules such as 1,3,5-trinitroperhydro-1,3,5-triazine (RDX)^38^. Toma *et al*. showed that engineered arrays of rectangular nanoantennas coupled through narrow gaps allow THz spectroscopy of a monolayer of CdSe quantum dots^40^. These impressive successes invites one to add to the arsenal of methods of THz field enhancement. Not only methods to achieve large field enhancements, but also simple and cheap methods deserve attention.

**Figure 1:**
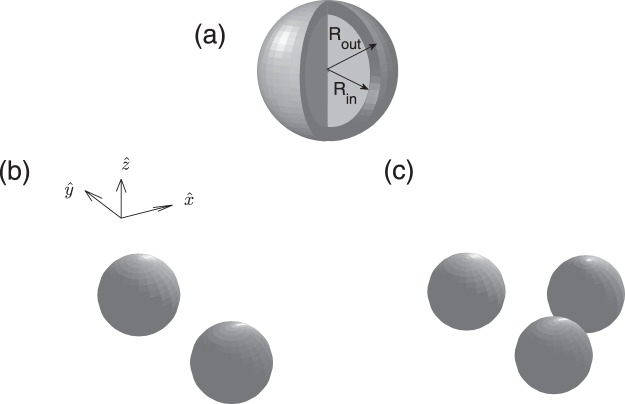
Schematic representation of (**a**) one, (**b**) two, and (**c**) three hollow InSb microspheres.

Indium antimonide (InSb) whose plasma frequency is in the THz range, has been widely used as a THz material^37,44–46^. Here we study electric and magnetic hotspots in the gap between hollow InSb microspheres forming dimers and trimers (see Fig. 1). The outer radius, core volume fraction, distance, and temperature of the microspheres can be chosen to address the necessity of intensity enhancement at a certain frequency corresponding to transition between energy levels of a molecule placed in the gap. For example, utilizing 80 *μ*m radius spheres at a gap of 2 *μ*m held at a temperature of 295 K, allow electric field intensity enhancements of 10–2880 and magnetic field intensity enhancements of 3–61 in the frequency window 0.35–1.50 THz. Here the core volume fraction and the ambient temperature influence the intensity enhancements, particularly in the frequency window 1.5–2 THz. Electric and magnetic hotspots provided by InSb microspheres are promising for THz absorption and circular dichroism spectroscopy.

## Model

We consider dimers and trimers of hollow InSb microspheres (see Fig. 1). We characterize the *α*th hollow microsphere by its position **r**_*α*_, inner radius *R*_in_, and outer radius *R*_out_. We use *f* = (*R*_in_/*R*_out_)^3^ to denote the core volume fraction. The permittivity of InSb can be well described by the Drude model^47^

1 $$\varepsilon (\omega ,T)={\varepsilon }_{\infty }-\frac{{\omega }_{p}^{2}(T)}{{\omega }^{2}+i\omega \gamma (T)}$$

where *ω*, *T*, *ε*_∞_, *ω*_*p*_, and *γ* are angular frequency, ambient temperature, high-frequency permittivity, plasma frequency, and damping factor, respectively. *ε*_∞_ = 15.75. ${\omega }_{p}(T)=\sqrt{4\pi {n}_{c}(T){e}^{2}/{m}^{\ast }}$, where the intrinsic carrier density is^48^

2 $${n}_{c}(T)=5.76\times {10}^{14}{T}^{1.5}\,\exp (\,-\frac{0.129\,e{\rm{V}}}{{k}_{B}T})\,{{\rm{cm}}}^{-3}$$

*e* is the charge of electron, *m*_*e*_ is the mass of electron, m* = 0.015*m*_*e*_ is the effective mass, and *k*_*B*_ is the Boltzmann constant. In particular^49^

3 $$\begin{array}{rcl}(\frac{{\omega }_{p}}{2\pi },\frac{\gamma }{2\pi }) & = & (8.0,0.26)\,{\rm{THz}}\,{\rm{at}}\,T=295\,{\rm{K}}\\ (\frac{{\omega }_{p}}{2\pi },\frac{\gamma }{2\pi }) & = & (9.0,0.28)\,{\rm{THz}}\,{\rm{at}}\,T=310\,{\rm{K}}\\ (\frac{{\omega }_{p}}{2\pi },\frac{\gamma }{2\pi }) & = & (9.9,0.31)\,{\rm{THz}}\,{\rm{at}}\,T=325\,{\rm{K}}\end{array}$$

We assume that the incident fields are

4 $$\begin{array}{rcl}{{\bf{E}}}_{I} & = & {E}_{0}{\hat{{\bf{u}}}}_{I}{e}^{i{{\bf{k}}}_{I}\cdot {\bf{r}}-i\omega t}\\ {{\bf{H}}}_{I} & = & {H}_{0}{\hat{{\bf{k}}}}_{I}\times {\hat{{\bf{u}}}}_{I}{e}^{i{{\bf{k}}}_{I}\cdot {\bf{r}}-i\omega t}\end{array}$$

The amplitude *E*_0_, polarization unit vector ${\hat{{\bf{u}}}}_{I}$, wave vector **k**_*I*_, and angular frequency *ω* = 2*πν* characterize the incident wave. *H*_0_ = *E*_0_, ${\hat{{\bf{u}}}}_{I}\cdot {\hat{{\bf{k}}}}_{I}=0$ and *k* = |**k**_*I*_| = *ω*/*c*, where *c* is the velocity of light in vacuum. Following the theory developed in ref. ^30^, we calculate the electromagnetic field distribution in the vicinity of the hollow microspheres. We use cgs units.

## Results and Discussion

### Field enhancement via InSb dimers and trimers

We assume that two identical hollow InSb microspheres are at positions **r**_1_ = (0, *R*_out_ + *d*_*p*_/2, *h*_*p*_) and **r**_2_ = (0, −*R*_out_ − *d*_*p*_/2, *h*_*p*_). We study the field enhancement factors |*E*/*E*_0_| and |*H*/*H*_0_|, where *E* and *H* denote the amplitudes of the total electric and magnetic fields at the observation point. The frequency *ν*_*E*_ (*ν*_*H*_) where the electric (magnetic) field enhancement factor gains its maximum, is of particular interest. We study InSb spheres of outer radius 30, 50 and 80 *μ*m. We assume that *d*_*p*_ = 2 *μ*m and *h*_*p*_ = 1 nm.

**Figure 2:**
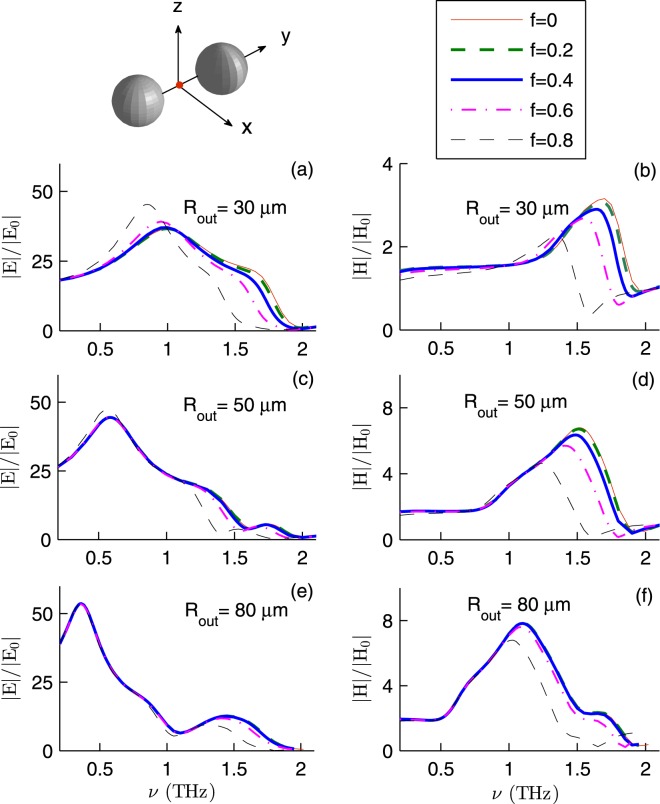
The field enhancement factors for InSb dimer as a function of the frequency *ν* for various *R*_out_ and *f*. Here *T* = 295 K, ${{\bf{E}}}_{I}\parallel \hat{{\bf{y}}}$, ${{\bf{k}}}_{I}=-\,k\hat{{\bf{z}}}$, and the fields are calculated at the dimer center (**r**_1_ + **r**_2_)/2.

Figure 2 shows the enhancement factors of InSb dimer. Here *T* = 295 K, ${{\bf{E}}}_{I}\parallel \hat{{\bf{y}}}$, that is, the incident electric field is parallel with the dimer axis, ${{\bf{k}}}_{I}=-\,k\hat{{\bf{z}}}$, and the fields are calculated at the dimer center (**r**_1_ + **r**_2_)/2. Quite remarkably, |*E*/*E*_0_| as large as (45.35, 47.36, 53.67) at frequency *ν*_*E*_ = (0.85, 0.57, 0.36) THz is achievable with spheres of outer radius (30, 50, 80) *μ*m and core fraction (0.8, 0.8, 0). Moreover, |*H*/*H*_0_| as large as (3.15, 6.70, 7.85) at frequency *ν*_*H*_ = (1.70, 1.51, 1.10) THz is achievable with spheres of outer radius (30, 50, 80) *μ*m and core fraction (0, 0 and 0.2, 0 and 0.2). In the case of 30 *μ*m radius spheres, *ν*_*E*_ slightly depends on *f*. Indeed *ν*_*E*_ shifts from 1.0 to 0.85 THz as *f* increases from 0 to 0.8. However, for 50 and 80 *μ*m radius spheres, *ν*_*E*_ does not depend on *f*. On the other hand *ν*_*H*_ shows a more clear dependence on *f*. For 50 *μ*m radius spheres, *ν*_*H*_ shifts from 1.51 to 1.25 THz as *f* increases from 0 to 0.8. Note that to obtain the largest enhancement at a certain frequency, the outer radius and core fraction must be chosen deliberately. For example at frequency 1.5 THz, 50 *μ*m spheres of core fraction 0.4 are superior to those of core fraction 0.8. At frequency 1 THz, 30 *μ*m radius spheres are superior (inferior) to 80 *μ*m radius ones in enhancing the electric (magnetic) field.

The *polarization* of the incident wave has a profound effect on the dimer optical response: The enhancement factors at the dimer center are not considerable when the incident electric field is perpendicular to the dimer axis.

**Figure 3:**
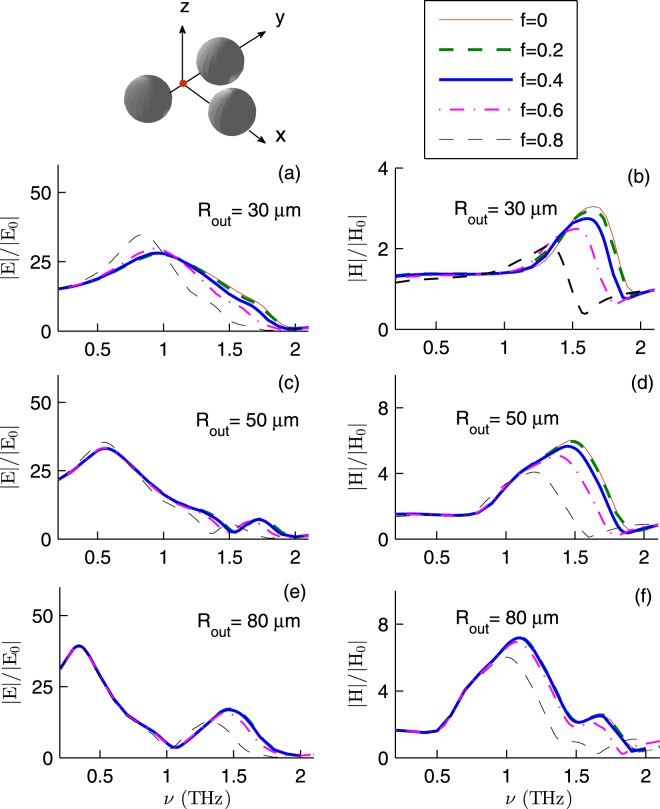
The field enhancement factors for InSb trimer as a function of *ν* for various *R*_out_ and *f*. Here *T* = 295 K, ${{\bf{E}}}_{I}\parallel \hat{{\bf{y}}}$, ${{\bf{k}}}_{I}=-\,k\hat{{\bf{z}}}$, and the fields are calculated at (**r**_1_ + **r**_2_)/2.

Now we pay attention to the symmetric trimer shown in Fig. 1. We assume that identical hollow microspheres are at **r**_1_, **r**_2_, and ${{\bf{r}}}_{3}=(\sqrt{3}{R}_{out}+\sqrt{3}{d}_{p}/2,0,{h}_{p})$. As before we assume that *d*_*p*_ = 2 *μ*m and *h*_*p*_ = 1 nm. Figure 3 shows the enhancement factors of InSb trimers. Here *T* = 295 K, ${{\bf{E}}}_{I}\parallel \hat{{\bf{y}}}$, ${{\bf{k}}}_{I}=-\,k\hat{{\bf{z}}}$, and the fields are calculated at (**r**_1_ + **r**_2_)/2. At first glance, Fig. 3 is not qualitatively different from Fig. 2. But upon a closer inspection, it becomes evident that the trimer may be used to *fine tune* the frequencies *ν*_*E*_ and *ν*_*H*_. Here |*E*/*E*_0_| as large as (34.73, 35.38, 39.37) at frequency *ν*_*E*_ = (0.83, 0.55, 0.34) THz is possible with spheres of outer radius (30, 50, 80) *μ*m and core fraction (0.8, 0.8, 0). Moreover, |*H*/*H*_0_| as large as (3.04, 6.02, 7.21) at frequency *ν*_*H*_ = (1.65, 1.49, 1.09) THz is possible with spheres of outer radius (30, 50, 80) *μ*m and core fraction (0, 0 and 0.2, 0 and 0.2). In the case of 80 *μ*m radius spheres, the right shoulder of |*E*/*E*_0_| is also of use. Here |*E*/*E*_0_| = (17.12, 17.13, 16.90, 15.70, 12.76) at frequency (1.46, 1.47, 1.47, 1.43, 1.31) THz when *f* = (0, 0.2, 0.4, 0.6, 0.8). Note that in a certain frequency window, the trimer may better act than the dimer. For example in the case of 80 *μ*m radius spheres of core fraction 0.8, the trimer better enhances the electric (magnetic) field in the frequency window 1.10–2 THz (1.44–2 THz).

We have also studied the field enhancement at the centroid of the trimer, assuming that *T* = 295 K, ${{\bf{E}}}_{I}\parallel \hat{{\bf{x}}}$ or ${{\bf{E}}}_{I}\parallel \hat{{\bf{y}}}$, and ${{\bf{k}}}_{I}=-\,k\hat{{\bf{z}}}$. We find that the electric (magnetic) field at (**r**_1_ + **r**_2_ + **r**_3_)/3 is about a factor ten (three) weaker than that at (**r**_1_ + **r**_2_)/2. Indeed with spheres of outer radius (30, 50, 80) *μ*m and core fraction (0.8, 0.8, all values of f), the maximum electric field enhancement (4.65, 3.63, 3.13) occurs at frequency *ν*_*E*_ = (0.83, 0.55, 0.33) THz. With spheres of outer radius (30, 50, 80) *μ*m and core fraction (0, 0, all values of f), the maximum magnetic field enhancement (1.55, 2.25, 2.19) occurs at frequency *ν*_*H*_ = (1.63, 1.45, 1.10) THz.

### The influence of ambient temperature on the field enhancement

**Figure 4:**
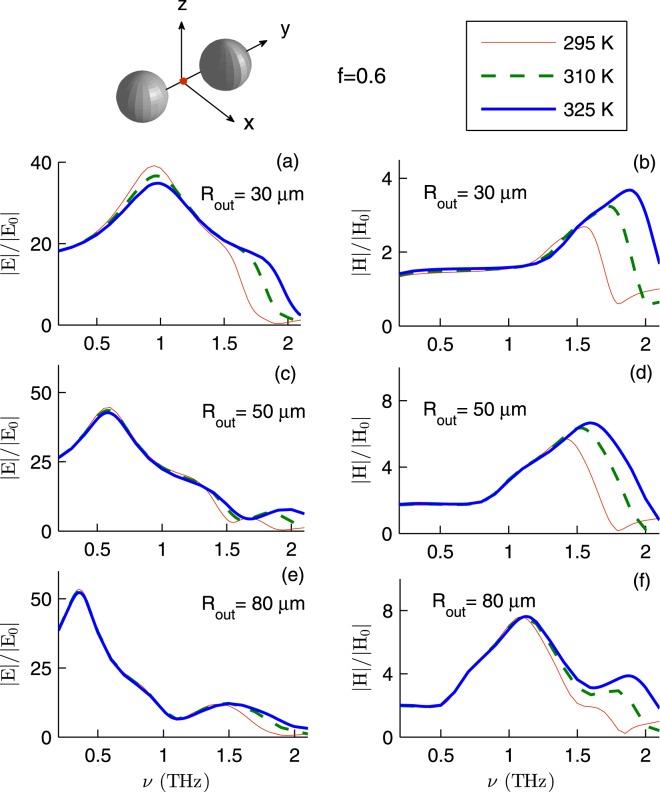
The field enhancement factors for InSb dimer as a function of *ν* for various *R*_out_ and *T*. Here *f* = 0.6, ${{\bf{E}}}_{I}\parallel \hat{{\bf{y}}}$, ${{\bf{k}}}_{I}=-\,k\hat{{\bf{z}}}$, and the fields are calculated at (**r**_1_ + **r**_2_)/2.

The plasma frequency *ω*_*p*_ and the damping factor *γ* of InSb depend on the ambient temperature *T*. Naturally, the ambient temperature influences the hotspots provided by InSb microspheres. Figure 4 exemplifies the enhancement factors for InSb dimer for three different temperatures 295, 310 and 325 K. Here *f* = 0.6, ${{\bf{E}}}_{I}\parallel \hat{{\bf{y}}}$, ${{\bf{k}}}_{I}=-\,k\hat{{\bf{z}}}$, and the fields are calculated at (**r**_1_ + **r**_2_)/2. We find that the electric and magnetic field enhancement factors of (30, 50, 80) *μ*m radius spheres show no clear temperature-dependence for frequencies lower than (0.50, 1.30, 1.49) THz and (1.20, 1.40, 1.10) THz, respectively. In the frequency window 1.5–2 THz, the enhancement factors increase as the temperature increases. For example, at frequency 1.80 THz the magnetic field enhancement of 50 *μ*m radius spheres increases from 0.16 to 5.15 as temperature increases from 295 to 325 K.

### The constructive interference of waves at the heart of the hotspots

**Figure 5:**
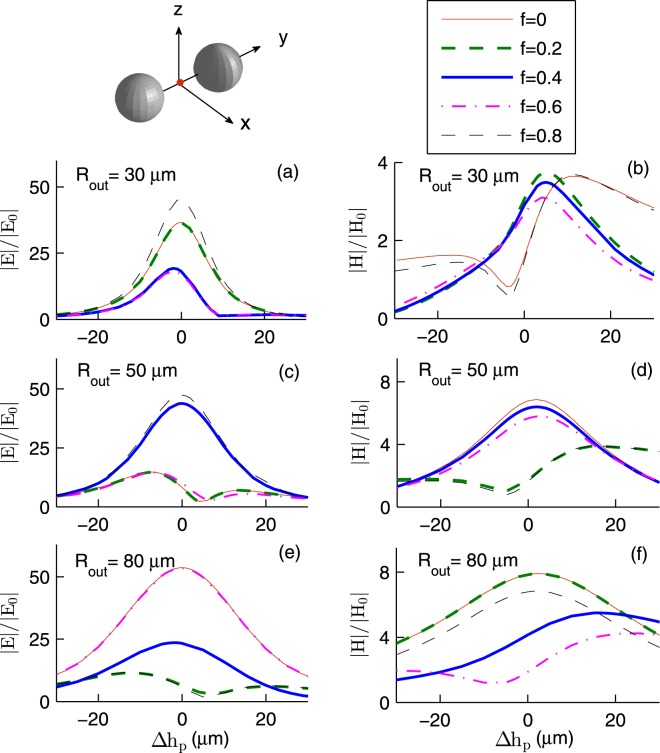
The field enhancements at the point $({{\bf{r}}}_{1}+{{\bf{r}}}_{2})/2+{\rm{\Delta }}{h}_{p}\hat{{\bf{z}}}$ as a function of Δ*h*_*p*_. Here *T* = 295 K, ${{\bf{E}}}_{I}\parallel \hat{{\bf{y}}}$, and ${{\bf{k}}}_{I}=-\,k\hat{{\bf{z}}}$. When *f* = (0, 0.2, 0.4, 0.6, 08) the frequency in THz is (**a**) (1.0, 0.99, 1.63, 1.55, 0.85), (**b**) (1.0, 1.67, 1.63, 1.55, 0.85), (**c**) (1.51, 1.51, 0.59, 1.41, 0.57), (**d**) (1.51, 0.59, 1.49, 1.41, 0.57), (**e**) (0.36, 1.1, 0.7, 0.36, 1.03), and (**f**) (1.1, 1.1, 0.7, 0.36, 1.03).

**Figure 6:**
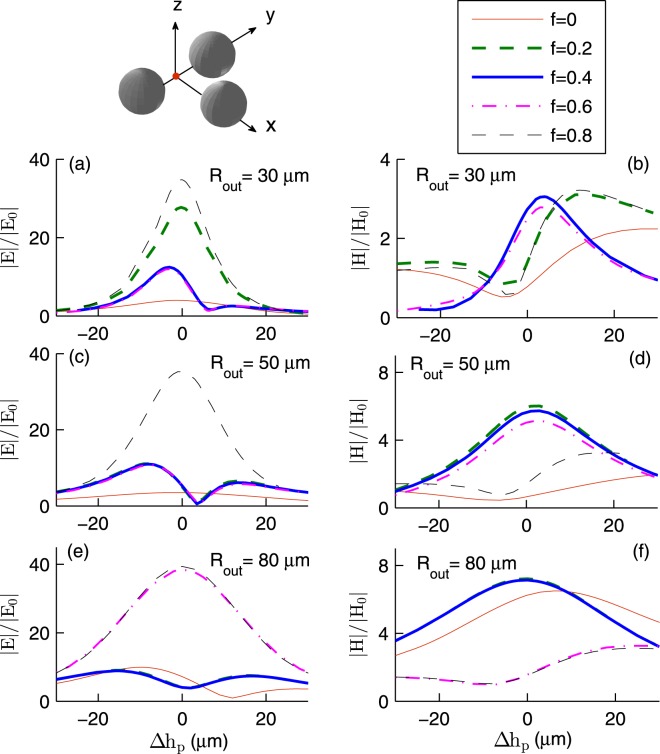
The field enhancements at the point $({{\bf{r}}}_{1}+{{\bf{r}}}_{2})/2+{\rm{\Delta }}{h}_{p}\hat{{\bf{z}}}$ as a function of Δ*h*_*p*_. Here *T* = 295 K, ${{\bf{E}}}_{I}\parallel \hat{{\bf{y}}}$, and ${{\bf{k}}}_{I}=-\,k\hat{{\bf{z}}}$. When *f* = (0, 0.2, 0.4, 0.6, 08) the frequency in THz is (**a** and **b**) (1.87, 0.97, 1.61, 1.53, 0.83), (**c** and **d**) (1.85, 1.47, 1.45, 1.4, 0.55), (**e** and **f**) (0.95, 1.1, 1.1, 0.35, 0.33).

At first thought, one may expect the maximum field enhancement to occur at the center of symmetry **r**_sym_ of a dimer or an equilateral trimer composed of similar microspheres. But the total field at a certain observation point **r**_obs_ is due to the *interference* of the incident field and the fields scattered by the microspheres. The InSb permittivity and hence the Mie scattering coefficients depend on the frequency of the incident field. Thus the multipole fields induced in the microspheres depend on the frequency and polarization of the driving field. For a certain frequency and polarization, the incident and the scattered fields may interfere more constructively at **r**_obs_ rather than at **r**_sym_. To confirm this, Figs 5 and 6 present field enhancements at a point displaced by the vector ${\rm{\Delta }}{h}_{p}\hat{{\bf{z}}}$ from the center of symmetry **r**_sym_. In many instances, the field reaches its maximum value when ${\rm{\Delta }}{h}_{p}\ne 0$. Here for specified parameters *ν*, *f*, *R*_out_, etc., the maximum electric (magnetic) field enhancement occurs at a point below (above) the center of symmetry. For example, in the case of solid 30 *μ*m radius spheres subject to 1.0 THz (1.87 THz) radiation, |*H*/*H*_0_| increases from 1.56 (0.78) to 3.65 (2.22) upon increasing the displacement from the center of the dimer (trimer) from zero to 10.5 *μ*m (25 *μ*m).

### The influence of core volume fraction on the field enhancements: The role of skin depth

As a first approximation, each microsphere can be modeled as a pair of an electric dipole and a magnetic dipole. The total electric (magnetic) field acting on a microsphere and the electric polarizability (magnetic polarizability) determine the induced electric (magnetic) dipole. Both electric and magnetic polarizabilities depend on the inner and outer radii of the microsphere. Thus one expects the field enhancement factors to depend on the core volume fraction of the InSb microspheres. The generalized multiparticle Mie theory also confirms this^30^. However, Figs 2–4 show that |*E*/*E*_0_| and |*H*/*H*_0_| *weakly* depend on *f* in the frequency window 0.35–1.50 THz. This can be understood if one reminds that the skin depth *δ*_*s*_, the distance over which the electromagnetic wave penetrates into the material, depends on the frequency *ν*.

**Figure 7:**
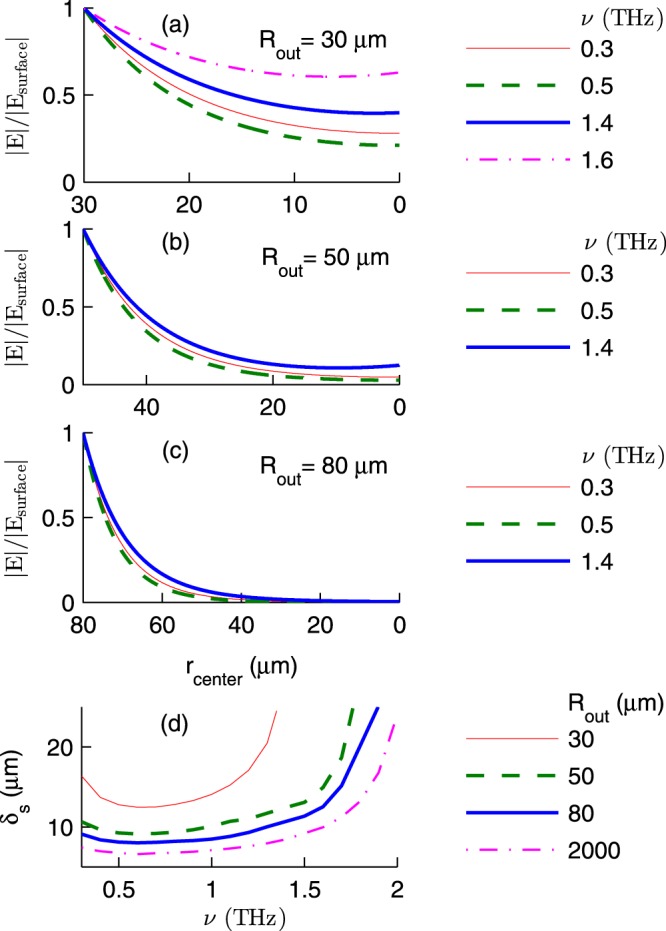
(**a**–**c**) |*E*/*E*_surface_| of a solid InSb microsphere as a function of *r*_center_ for various outer radii and frequencies. (**d**) The skin depth *δ*_*s*_ as a function of frequency *ν* for various outer radii. Here *T* = 295 K.

Let us consider *one* solid InSb microsphere, and denote the radial distance from its centre, and the magnitude of the electric field at its surface by *r*_center_ and |*E*_surface_|, respectively. Figure 7(a–c) demonstrate that for various outer radii and frequencies, |*E*/*E*_surface_| is a *monotonically* decreasing function of *r*_center_. Thus it is appropriate to introduce the skin depth *δ*_*s*_ where |*E*/*E*_surface_| is 1/*e* ≈ 0.368. Figure 7(d) shows extracted *δ*_*s*_ as a function of *ν* for various *R*_out_. For example, in the case of 80 *μ*m radius spheres, the skin depth is 8.46 *μ*m at 1.0 THz. Thus we anticipate that at frequency 1.0 THz, the optical response of solid and hollow microspheres with *R*_in_ < 71.54 *μ*m (*f* < 0.72) are almost the same. The skin depth increases from 11.33 to 25.55 *μ*m as frequency increases from 1.5 to 2.0 THz. Hence we anticipate that in the frequency window 1.5–2.0 THz, the core fraction strongly influences the enhancement factors. Our detailed calculations are in agreement with these anticipations (see Figs 2–4). Figure 7(d) also shows that the skin depths for 80 and 2000 *μ*m radius spheres are not much different. Now it is clear that using large InSb microspheres, one finds that *ν*_*E*_ and *ν*_*H*_ very weakly depend on *f*. The skin depth of a 30 *μ*m radius sphere needs a separate discussion: Fig. 7(a) shows |*E*/*E*_surface_| at frequencies 1.4 and 1.6 THz. Indeed *δ*_*s*_ is not well defined for *ν* > 1.35 THz where |*E*/*E*_surface_| > 0.368 at *r*_center_ = 0.

## Conclusions

A few remarks are in order. (i) Here we have considered microspheres at a gap of 2 *μ*m. We expect the enhancement factors |*E*/*E*_0_| and |*H*/*H*_0_| to increase as the gap decreases. (ii) Here we have considered dimers and trimers of identical microspheres. Microspheres of different outer radius, core volume fraction, distance, and composition may allow better field enhancement. (iii) Populous disordered and fractal clusters of InSb microspheres deserve a separate study. (iv) It is instructive to remind the maximum enhancement factors in the wavelength window 400–1100 nm that dimers and trimers of hollow nanoparticles provide. Ag particles of outer radius 80 nm and core volume fraction 0.8 at a gap of 10 nm, provide electric field intensity enhancement as large as 2538 at wavelength 1020 nm. Using Si particles of outer radius 120 nm and core volume fraction 0.4 at a gap of 10 nm, magnetic field intensity enhancement as large as 109 at wavelength 612 nm is achievable^30^. Our results show that the field enhancements of InSb microspheres in the THz region of the spectrum are comparable to those of Ag and Si nanoparticles in the visible and near-infrared regions of the spectrum. (v) As early as 1978, Chen and Furdyna^50^ prepared InSb microspheres by ultrasonically cutting small cylinders from a slab and abrading them in an air grinder. Recent successful fabrication of hollow InP microspheres^51–53^ suggests that facile and cheap preparation of hollow InSb microspheres is not out of reach. (vi) The *simplicity* of using InSb microspheres to obtain reasonable THz electric and magnetic field enhancement, can be considered as an advantage.

In summary, we have studied electric and magnetic hotspots in the gap between hollow InSb microspheres forming dimers and trimers. To achieve field enhancement at a certain frequency corresponding to transition between energy levels of a molecule placed in the gap, the outer radius, core volume fraction, distance, and temperature of the microspheres can be chosen. For example, at the center of a dimer composed of 80 *μ*m radius spheres at a gap of 2 *μ*m, electric field intensity enhancements of 10–2880 and magnetic field intensity enhancements of 3–61 in the frequency window 0.35–1.50 THz are achievable. Here the incident electric field is parallel with the dimer axis, and *T* = 295 K. The core volume fraction ($0\leqslant f\leqslant 0.8$) and the ambient temperature ($295\leqslant T\leqslant 325\,{\rm{K}}$) influence the intensity enhancements, particularly in the frequency window 1.5–2 THz. The enhancement factors are not considerable when the incident electric field is perpendicular to the dimer axis. Provided that all microspheres are fabricated with the same size, the maximum field enhancements presented by the trimer are less than those of the dimer. Nevertheless, in a certain frequency window, the trimer may be superior to the dimer. For instance using 80 *μ*m radius spheres of core fraction 0.2, the trimer better enhances the electric (magnetic) field in the frequency window 1.23–1.73 THz (1.59–1.75 THz). We have shown that the frequency dependence of the skin depth explains the weak dependence of the enhancement factors on the core volume fraction. Electric and magnetic hotspots provided by InSb microspheres are promising for THz absorption and circular dichroism spectroscopy.
